# The subcortical basis of subjective sleep quality

**DOI:** 10.1101/2024.05.29.596530

**Published:** 2024-06-04

**Authors:** Martin M. Monti

**Affiliations:** 1 Department of Psychology, University of California Los Angeles, 502 Portola Plaza, Los Angeles, 90095, CA, USA; 2 Brain Injury Research Center (BIRC), Department of Neurosurgery, University of California Los Angeles, 300 Stein Plaza Driveway, Los Angeles, 90095, CA, USA

**Keywords:** Sleep and Brain, Neuroanatomy, Neuroimaging, Thalamus, Globus Pallidus, Striatum

## Abstract

**Study objectives:**

To assess the association between self-reported sleep quality and cortical and subcortical local morphometry.

**Methods:**

Sleep and neuroanatomical data from the full release of the young adult Human Connectome Project dataset were analyzed. Sleep quality was operationalized with the Pittsburgh Sleep Quality Index (PSQI). Local cortical and subcortical morphometry was measured with subject-specific segmentations resulting in voxelwise thickness measurements for cortex and relative (i.e., cross-sectional) local atrophy measurements for subcortical regions.

**Results:**

Relative atrophy across several subcortical regions, including bilateral pallidum, striatum, and thalamus, was negatively associated with both global PSQI score and sub-components of the index related to sleep duration, efficiency, and quality. Conversely, we found no association between cortical morphometric measurements and self-reported sleep quality.

**Conclusions:**

This work shows that subcortical regions such as the bilateral pallidum, thalamus, and striatum, might be interventional targets to ameliorate self-reported sleep quality.

## Introduction

Highly conserved across the animal kingdom [1], sleep is important for cognitive, social, and emotional functioning [2–4] across the lifespan [5, 6]. Indeed, sleep deprivation has been associated with cognitive and emotional dysfunction [7–12], sleep disturbances are associated with psychiatric illnesses [13–16] and are part of the known non-motor symptomatology of multiple neurodegenerative disorders [17–22].

Despite the importance of sleep, our understanding of its relationship with brain morphology remains in the early phases [23, 24]. Furthermore, while large-sample studies are now starting to emerge [25–30], the results are yet to coalesce and remain variable, perhaps also due to different methodological choices [31], often with relatively small effect sizes despite increasingly sophisticated approaches. Yet, a greater understanding of the relationship between brain structures and sleep is key to devising new interventions to mitigate sleep-related disorders [32].

In what follows, we leverage the full release of the Human Connectome Project (HCP) [33], to assess the relationship between subjective sleep quality indicators [34] and brain morphometry using local (i.e., voxelwise) cortical [35, 36] and subcortical [37] morphometry, as opposed to the global and regional features (i.e., average regional volumes, areas, and thickness) used in prior work [27, 29, 30]. In particular, we highlight that while prior studies have leveraged a subset of the data we use for assessing functional and structural associations with subjective sleep quality [26, 38], the sensitive subcortical approach we apply [37] is entirely novel in this context. As described below, using this sensitive approach, we find systematic associations between the global subjective sleep quality and widespread subcortical atrophy.

## Methods

### Sample description

This project is based on the Q3 release of the Human Connectome Project (HCP; [33]). Out of the 1206 total participants, 93 could not be included in the analysis due to failure to acquire at least one T1-weighted MRI dataset, resulting in an included sample of 1,113 datasets. (As described below, one additional dataset was excluded due segmentation failure, resulting in a final analyzed sample of 1,112 datasets). The final analyzed sample included data from 605 female and 507 male, with 231 individuals in the 22–25 age group, 486 in the 26–30 age group, 382 in the 31–35 age group, and 13 individuals 36 years old or greater.

### PSQI data

For each participant, we collected the total score and the individual component scores of the Pittsburgh sleep Quality Index (PSQI; [34]) as made available in the most recent HCP data distribution. Because of extensive correlations across the seven components of the PSQI (see SOM1), prior to analyzing their relationship to brain structures, we performed a data reduction use a Principal Component Analysis (PCA; [39]) approach. The PCA was performed in JASP 2024 (v 0.18.3; [40]), using an orthogonal varimax rotation and retaining all components with an eigenvalue greater than 1. This data reduction resulted in two orthogonal components explaining 50% of the variance. As shown in Tab. 1, the first principal component loaded mainly on the sleep quality, sleep duration, and sleep efficiency PSQI components (with loadings equal to 0.62, 0.83, and 0.74, respectively). The second principal component loaded mainly on the sleep onset latency, sleep disturbances, and use of sleep medications PSQI components (with loadings equal to 0.67, 0.61, and 0.69, respectively). The daytime dysfunction PSQI component was loaded upon very weakly (< 0.36) by both PCs.

### MRI data

A total of 1,113 T1-weighted data were obtained from the HCP Young Adult repository. For this project we selected, for each participant, the “T1w_MPR1” dataset acquired as part of the “structural session”. As described in the HCP Q3 release appendix (available at http://www.humanconnectome.org) these data were acquired with a Magnetization-Prepared Rapid Acquisition Gradient Echo (MPRAGE) sequence (TR = 2,400 ms, TE = 2.14 ms, TI = 1,000 ms, GRAPPA acceleration factor = 2). The acquisition resulted in an image size of 256×320×243 voxels (in the x, y, and z directions, respectively) and an isovoxel resolution of 0.7 mm.

### MRI data analysis

#### T1-weighted data preprocessing and segmentation

**Fig. 1.**
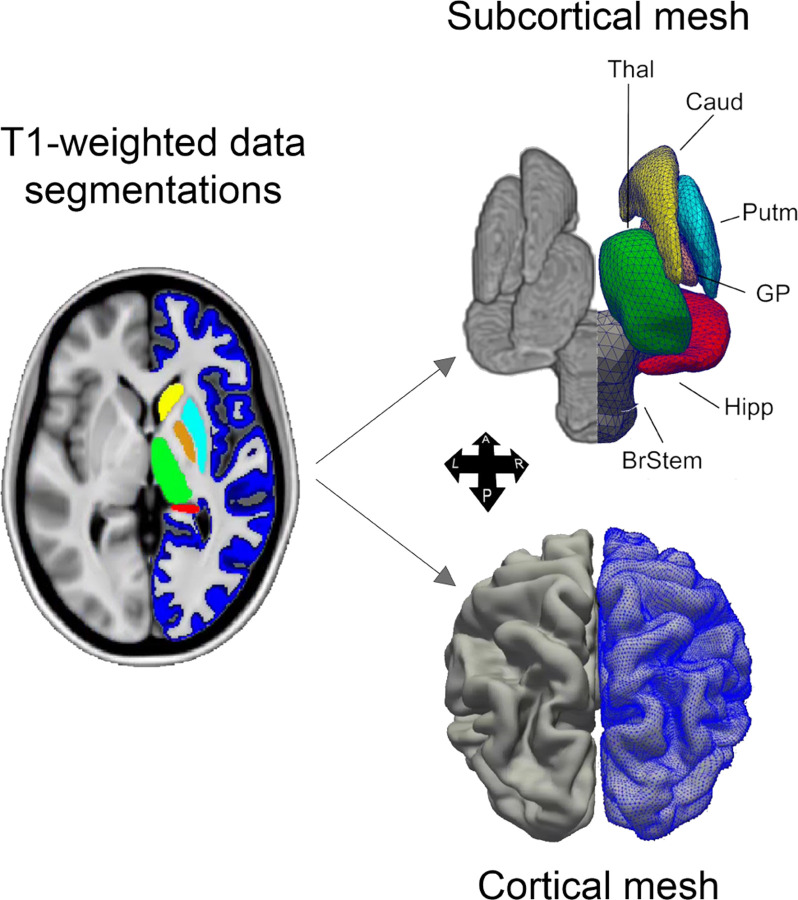
Methods. Left: Illustration of cortical and subcortical segmentations of the T1-weighted data. Right: Illustration of 3D subcortical (top) and cortical (bottom) reconstructed meshes. (Image partially adapted from [41])

All MRI data analyses were performed with the FMRIB Software Library (FSL; [42]). T1-weighted data were first preprocessed using the fsl_anat script, which provides a general pipeline for processing anatomical images. For each dataset, the script reorients images to the standard (MNI) orientation (fslreorient2std), crops the image (robustfov), performs bias-field correction (RF/B1-inhomogeneity-correction; FAST; [43]), registration to standard space (linear and non-linear; FLIRT and FNIRT, [44, 45]), brain-extraction (FNIRT-based), tissue-type segmentation (FAST; [43]), and subcortical structure segmentation (FIRST; [37]). Once subcortical segmentations were completed, and before any analysis, the resulting subcortical meshes were individually inspected by the author to ensure correct structure segmentation (cf., Fig. 1). During this control, it emerged that segmentation for one dataset failed, not producing any mesh output. That dataset was thus excluded from any further analysis (leading to a final analyzed sample of 1,112 datasets). Cortical segmentations were obtained by feeding the bias-corrected T1-weighted data to FSL Voxel Based Morphometry (VBM; [46]) scripts, an optimized VBM protocol [36] enclosed in the FSL distribution. Again, segmentations were visually inspected for accuracy prior to any analysis. All 1,112 datasets return satisfactory segmentations of the cortical gray matter (cf., Fig. 1).

#### T1-weighted data analysis

The obtained single subject cortical segmentations and subcortical meshes were entered into a group VBM analysis and a group shape (or vertex) analysis, respectively. While both cortical and subcortical tissues are segmented in the subject’s native space, subcortical meshes are then co-registered to the standard MNI space for the purposes of group analysis. Conversely, cortical segmentations are co-registered to an *ad-hoc* sample-specific template created, automatically, as an average of all the 1, 112 input data, for the purposes of performing the VBM group analysis. Each set of segmentations (i.e., cortical, subcortical) were entered into two group analyses. First, we regressed the segmentations on the PSQI total score (henceforth, PSQI total score analysis). Second, we regressed the segmentations the two PCA components described above (henceforth, PSQI PCA analysis). In addition to these regressors of interest, each of these two statistical models also included sex and age-group covariates, as well as each subject’s total normalized brain volume (as calculated with SIENAX; [47]) in order to account for the effect of head size variability across individuals and to ensure that results reflect local shape change [41, 48].

Group analyses were performed with Randomise [49] using a non-parametric permutation testing approach [50]. Significance was assessed against a criterion of *α* ≤ 0.05 corrected for multiplicity with a family-wise cluster correction approach with 2-dimensional Threshold Free Cluster Enhancement (TFCE; [51, 52]).

## Results

### PSQI data analysis

**Fig. 2.**
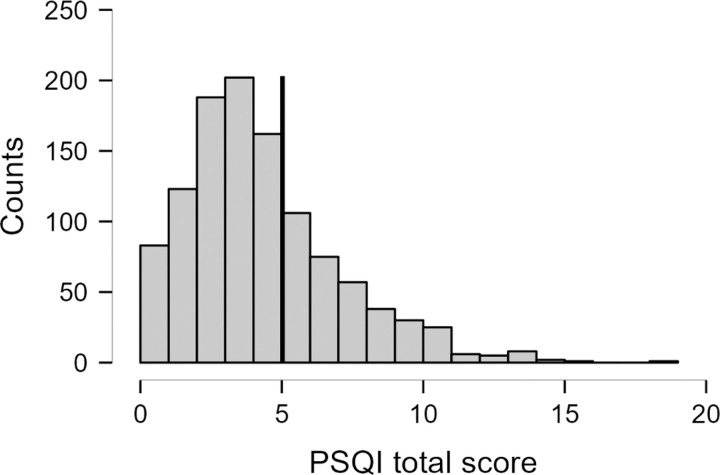
Distribution of PSQI total scores in the analyzed sample. The black line indicates the conventional criterion discriminating “bad sleepers” (i.e., PSQI total score > 5) from “good sleepers” (i.e., PSQI total score ≤ 5; [34]).

As shown in Fig. 2 and in Tab. 1, the PSQI total score in the analyzed sample had a mean of 4.8 and a median of 4. Using the conventional criterion of PSQI 5 [34], 38% of participants in the analyzed sample were classified as having poor sleep quality. The proportion of poor sleepers was not significantly different across male and female participants (i.e., 31% and 33% for male and female, respectively; *χ*^2^(1) = 0.685, p= 0.408) nor across the four age groups (i.e., 33%, 33%, 31%, and 21% for the 22–25, 26–30, 31–35, and over 35 age groups, respectively; *χ*^2^(3) = 0.932, p= 0.818).

### MRI results

**Fig. 3.**
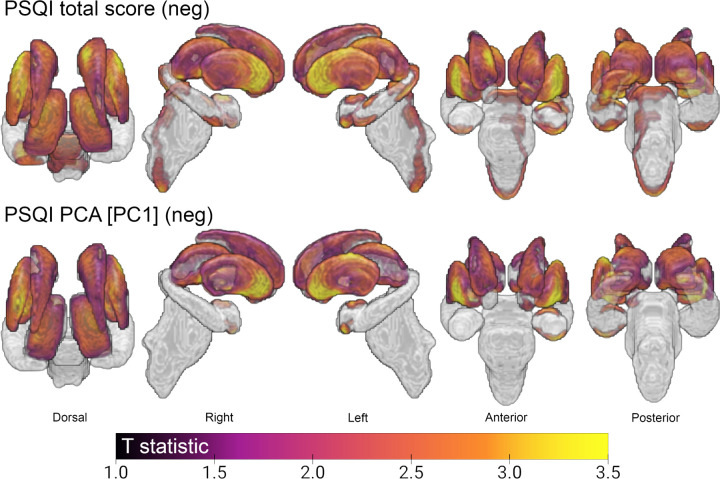
Subcortical results. Areas significantly associated, negatively, with the PSQI total score analysis (top) and the PSQI PCA analysis (PC1). (Areas shown in color indicate significant voxels. Areas shown in gray imply no significant result.)

As shown in Fig. 3 and Tab. 2 (see also SOM2), the PSQI total score analysis resulted in extensive negative correlations with many subcortical regions. Specifically, extensive negative associations were found between the PSQI total score and atrophy in the bilateral pallidum (99.2% and 97.6% of left and right hemisphere voxels, respectively), thalamus (96.1% and 99.3% of left and right hemisphere voxels, respectively), putamen (91.3% and 89.1% of left and right hemisphere voxels, respectively), and caudate (64.7% and 83.0% of left and right hemisphere voxels, respectively). Additionally, regions of significant negative association with the PSQI total score were also observed in the left nucleus accumbens and amygdala (64.7% and 45.8% of voxels, respectively), bilateral hippocampus (27.1% and 6.7% of left and right hemisphere voxels, respectively), and in the brainstem (20.2% of voxels). No positive correlations were detected between PSQI total score and subcortical shape. Similarly, no associations were found, either positive or negative, with cortical thickness.

The PSQI PCA analyses returned significant negative associations between PC1 and most of the regions found in the PSQI total score analysis regions (cf., Fig. 3 and Tab. 2). Specifically, significant negative associations were observed in the bilateral pallidum (91.1% and 89.8% of left and right hemisphere voxels, respectively), putamen (86.0% and 82.3% of left and right hemisphere voxels, respectively), caudate (66.9% and 82.3% of left and right voxels, respectively), and thalamus (67.6% and 82.3% of left and right hemisphere voxels, respectively). Smaller areas of significant negative association with PC1 were also uncovered in the left nucleus accumbens (35.6% of voxels) and amygdala (32.3% of voxels), and right hippocampus (1.6% of voxels). No positive associations were found between PC1 and subcortical local morphometry. Similarly, no associations were found, either positive or negative, between subcortical local morphometry and PC2. Mirroring the first analysis, no associations were found, either positive or negative, between local cortical measurements and either PC.

Given the null voxelwise cortical results, in order to assess whether this was due to the specific analytical protocol employed [53], we leveraged the existing global, cortical and subcortical morphometric analyses made available as part of the HCP data distribution (and comparable to some of the measurements discussed in prior work [29, 30]). As described elsewhere [54], this volumetric analysis is based on a different software than the analysis reported above (i.e., FreeSurfer version 5.2 [55, 56], as opposed to FSL) and a different processing pipeline (i.e., the so-called HCP minimal processing structural pipeline [54], obtained by running three scripts included in FreeSurfer : *PreFreeSurfer*, *FreeSurfer*, and *PostFreeSurfer*). When we performed a Mann-Whitney independent samples test (due to violation of normality, as assessed with a Shapiro-Wilk test [57]) comparing “good sleepers” versus “bad sleepers,” only two out of the 199 global and region-specific morphometric measurements available exhibited a significant difference between the two groups (using the Benjamini Hochberg FDR adjustment for multiplicity [58]): cortical thickness in the right entorhinal and temporal pole cortices (cf., Table SOM1). Beyond that, we found no association between any of the remaining global and local (including regional thickness, area, and volume) measurement and self-reported sleep quality (cf., Table SOM1).

## Discussion

In the present work, we report two main results. First, we show that sleep quality, as assessed by the PSQI total score, as well as by specific PSQI components (i.e., quality, duration, and efficiency; cf., 1), are associated with significant inwards displacement of several subcortical areas including, in particular, the bilateral globus pallidus, thalamus, and the striatum. Conventionally, this result is interpreted as indicating that worse sleep quality is associated with local atrophy in these regions [37]. Associations between subcortical regions and sleep have a long history in the context of pre-clinical animal models [59, 60]. Only more recently this association has returned to the forefront [32, 61, 62]. In particular, our results show maximal association between the PSQI measures (total, as well as PC1) and bilateral globus pallidus (cf., Fig. SOM), striatum, and thalamus.

Thalamus, and its interactions with cortex, have long been implicated in sleep-wake transitions, as a key target of ascending arousal system [63, 64]. Indeed, sleep disturbances are associated with thalamic abnormalities and/or dysfunction across a large set of mental health and neuropsychiatric disorders. In patients with insomnia, for example, clinical features of the pathology are associated with thalamic structural and functional abnormalities [65, 66]. Aberrant metabolite levels in thalamus have been found, in multiple diseases, to be associated negatively with sleep quality measurements [67, 68]. Furthermore, long-term sleep deprivation is known to be associated with decreased thalamic volume [69], while thalamic stroke and infarction are known to lead to sleep impairments [70, 71]. Even in healthy volunteers, across the lifespan, thalamic volumes and function are associated with sleep disturbances [72]. Our finding of an association between thalamic atrophy and PSQI does stand in contrast to some prior reports [73]. While we can only speculate on the reasons for the inconsistency, it is possible that the use of a much larger sample (i.e., 1,112 versus 54), including a larger age range, and a more sensitive analysis (i.e., local vertex displacement versus global thalamic volume) might explain, to some extent, the difference in findings.

Long known to serve a key role in the control of motor behavior [74], the contributions of the basal ganglia to non-motor function have only come to the forefront relatively recently [61, 62], together with a renewed understanding of the complex circuitry tying these regions together and within broader cortical-subcortical circuits [32, 75, 76]. Indeed, clinical data show that patients with striatal dysfunction syndromes, such as Parkinson’s and Huntington Disease, exhibit high rates of sleep disturbances [17–20, 77], something that is likely to be mediated, at least in part, by dopamine mechanisms [78]. Interestingly, the availability of striatal dopamine in D2/D3 receptors has been associated with sleep quality in healthy adults [79]. Consistent with this view, and with our findings in healthy volunteers, lesion studies in the rodent model have documented alteration of sleep-wake behavior following striatal damage [60, 80]. In particular, striatal lesions have been shown to lead to an increased number of state transitions and loss of ultra-long wake bouts (i.e., greater sleep fragmentation) [81]. In addition, the role of dopamine in modulating sleep-wake behavior might rely on the action of a number of subcortical regions, including the nucleus accumbens [81–83], a regions which we also find to be atrophic in individuals with poorer sleep quality. It is not surprising, then, that despite a certain heterogeneity in findings, self-reported sleep has been associated multiple time to neuroanatomical measurements in these regions [29, 30].

The region most extensively associated with our measures of sleep quality (i.e., PSQI total score and PSQI PC1) was the bilateral globus pallidus. Traditionally conceived as a relay in the control of motor behavior [84–86], a growing amount of evidence is linking this region – and its external portion in particular – to the regulation of sleep-wake behavior and electrophysiology [61, 62], likely *via* dopaminergic neuromodulation [87]. Indeed, as found with the striatum, excitotoxic lesions of the external portion of the pallidum, in rodent models, increase sleep fragmentation, with increased sleep-wake transitions and decreased sleep bouts [81, 88]. Conversely, optogenetic and electical neuromodulation of this region results in a broad increase of time spent sleeping [88, 89]. Consistent with these findings, in humans, the pallidum has been associated to behavioral and/or electrocortical arousal in healthy volunteers undergoing general anesthesia [90], and in severe brain injury patients [41, 48]. Intriguingly, and mirroring the rodent work, deep brain stimulation of the external portion of the pallidum in a parkinsoninan patient dramatically improved their sleep [91].

Finally, although to a much smaller extent than the regions discussed above, we also found systematic associations between worse subjective sleep quality and amygdalar and hippocampal atrophy. With respect to the latter, prior work suggests a negative relation between hippocampal morphometry and self-reported sleep indices both cross-sectionally [30] and longitudinally [27]. Furthermore, we highlight that, considering the relatively small effect sizes reported in prior work, the fact that we uncover a similar association, in a small portion of the hippocampus, in a different sample, and with a different methodology, offers concordant evidence for hippocampus playing a role in explaining individual differences in self-reported sleep. With respect to the amygdala, our findings are consistent with prior reports associating amygdalar atrophy, connectivity dysfunction, and sleep disturbances across a number of conditions [30, 92–95], dissociably from its comorbid association with anxiety [96].

Our second main finding is that, in stark contrast to the subcortical results, we find no association between voxelwise cortical thinning and self-reported measures of sleep quality. This general result agrees with recent reports [29, 97], including one performing a similar analysis (though with different methodology) in a subset of the sample we used [26]. Nonetheless, these results do stand in contrast with other prior reports [30, 98, 99]. Interestingly, even when we leveraged the HCP-provided regional (and global) morphometric measurements, which make use of a methodology similar to published work [30, 98], we find only 2 significant associations with subjective sleep quality and uncover overwhelmingly small effect sizes (cf., Tab. SOM1). Furthermore, it should also be highlighted that, so far, the few significant cortical associations reported across studies are heterogeneous [30, 98, 99]. It remains to be resolved in future work whether the variance in cortical results is genuine or reflects methodological discrepancies [31] and/or the known greater variability in cortical, versus subcortical, neuroanatomy [100].

In assessing the present findings, one must take stock of a number of limitations tied to the analytical approach employed. First, the segmentation technique we used [37] cannot capture a number of important regions and subregions that are believed to play an important role in the regulation of sleep-wake behavior [101, 102]. Second, our morphometric approach centers on local cortical and subcortical structures, but is blind to other factors (e.g., white matter integrity, glymphatic system), which have been proposed to play a role in sleep [97, 103]. Finally, this work is focused entirely on neuroanatomical variability, yet there exist known associations between changes in brain function and sleep quality [38, 104–107]. It will be interesting, in future work, to delineate the degree to which (if any) the known associations between brain network function and perceived sleep quality [38, 108] is related to the neuroanatomical variability described in the present work.

## Conclusion

Overall, the present work shows that subjective sleep quality, as captured by conventional self-report [34], in the general population is directly related to the shape of subcortical nodes and, in particular, the globus pallidus, striatum, and thalamus. Importantly, we show that it is not necessarily the overall volume of these regions that is associated with sleep quality, but rather the specific topological focus of thinning/atrophy. This work shows that subcortical regions, including the globus pallidus, thalamus, and the striatum, might be potential targets to ameliorate sleep in otherwise healthy individuals and in a large variety of mental health and neuropsychiatric disorders [32].

## Supplementary Material

Supplement 1

## Figures and Tables

**Table 1.** Characteristics of the PSQI total score and each component, for the analyzed sample. (Abbreviations: Std. Dev., standard deviation; Min., minimum; Max., maximum; PC, principal component; Tot., total; sleep qual., sleep quality; sleep ons. lat., sleep onset latency; sleep dur., sleep duration; sleep effic., sleep efficiency; sleep disturb., sleep disturbances; use of sleep med., use of sleep medications; daytime dysf., daytime dysfunction.)

PSQI	Mean	Std. Dev.	Min.	Max.	PC
Tot. Score	4.80	2.76	0	19	
*PSQI comp.*					
Subj. sleep qual.	0.89	0.63	0	3	1
Sleep ons. lat.	0.97	0.82	0	3	2
Sleep dur.	0.57	0.82	0	3	1
Sleep effic.	0.46	0.79	0	3	1
Sleep distrub.	1.09	0.49	0	3	2
Use of sleep med.	0.24	0.68	0	3	2
Daytime dysf.	0.59	0.64	0	3	-

**Table 2.** Subcortical results. Proportion of significant voxel for each subcortical structure. (Abbreviations: Hem., hemisphere; Prop. Sig. Voxels, Proportion of Significant Voxels; L, left; R, Right)

Structure	Hem.	Prop. Sig. Voxels (%)
*PSQI total score analysis (negative association)*		
Thalamus	R	99.27%
Pallidum	L	99.19%
Pallidum	R	97.62%
Thalamus	L	96.10%
Putamen	L	91.32%
Putamen	R	89.07%
Caudate	R	83.04%
Nucleus Accumbens	L	64.71%
Caudate	L	64.08%
Amygdala	L	45.77%
Hippocampus	L	27.06%
Brain Stem		20.21%
Hippocampus	R	6.67%
Nucleus Accumbens	R	0.00%
Amygdala	R	0.00%
*PSQI PCA analysis PC1 (neg. association)*		
Pallidum	L	91.05%
Pallidum	R	89.77%
Putamen	L	86.03%
Caudate	R	82.34%
Thalamus	R	82.28%
Putamen	R	75.82%
Thalamus	L	67.61%
Caudate	L	66.91%
Nucleus Accumbens	L	35.85%
Amygdala	L	32.36%
Hippocampus	R	1.57%
Brain Stem		0.00%
Hippocampus	L	0.00%
Nucleus Accumbens	R	0.00%
Amygdala	R	0.00%

## Data Availability

The data underlying this article are publicly available at https://www.humanconnectome.org/study/hcp-young-adult. This manuscript is made available on BiorXiv as preprint https://doi.org/10.1101/2024.05.29.596530.

## Statement of Significance

In this study we address, with a novel sensitive approach, the relationship between subjective sleep quality and neuroanatomy in a large population of young adults. We find that measures of subjective sleep quality are inversely related to atrophy in deep brain nuclei including the bilateral pallidum, striatum, and thalamus. These results provide potential intervention targets for mitigating sleep-related disorders and improving sleep quality.
